# Zoonotic and reverse zoonotic events of SARS-CoV-2 and their impact on global health

**DOI:** 10.1080/22221751.2020.1827984

**Published:** 2020-10-14

**Authors:** Khalid Munir, Shoaib Ashraf, Isra Munir, Hamna Khalid, Mohammad Akram Muneer, Noreen Mukhtar, Shahid Amin, Sohaib Ashraf, Muhammad Ahmad Imran, Umer Chaudhry, Muhammad Usman Zaheer, Maria Arshad, Rukhsana Munir, Ali Ahmad, Xin Zhao

**Affiliations:** a PetLife Veterinary Professional Corporation, NJ, USA; b Department of Pathobiology, Riphah College of Veterinary Sciences, Riphah University, Lahore, Pakistan; c Wellman Center for Photomedicine, Massachusetts General Hospital, Harvard Medical School, Boston, MA, USA; d School of Dental Medicine, University of Pennsylvania, Philadelphia, PA, USA; e Department of Chemical and Biological Engineering, Villanova University, Villanova, PA, USA; f Animal Hospital of Loves Park, Loves Park, IL, USA; g Department of Microbiology, Shaikh Zayed Hospital Lahore, Lahore, Pakistan; h ; Royal (Dick) School of Veterinary Studies and Roslin Institute, Edinburgh, UK; i Food and Agriculture Organization of the United Nations, Country Office, Islamabad, Pakistan; j District Headquarter Hospital, Lahore, Pakistan; k Consultant Emergency Medicine, Russells Hall Hospital, Dudley Group of Hospitals NHS Trust, Dudley, UK; l CHU Sainte-Justine Research Center, Department of Microbiology, Infectious Diseases and Immunology, University of Montreal, Montreal, Canada; m Department of Animal Sciences, McGill University, Sainte-Anne-de-Bellevue, Canada

**Keywords:** Coronavirus, COVID-19, reverse zoonosis, SARS-CoV-2, secondary zoonosis, One-Health One-World, zoonosis

## Abstract

Coronaviruses (CoVs) are enveloped, positive sense, single-stranded RNA viruses. The viruses have adapted to infect a large number of animal species, ranging from bats to camels. At present, seven CoVs infect humans, of which Severe Acute Respiratory Syndrome Coronavirus-2 (SARS-CoV-2) is responsible for causing the Coronavirus Disease 2019 (COVID-19) in humans. Since its emergence in late 2019, SARS-CoV-2 has spread rapidly across the globe. Healthcare systems around the globe have been stretched beyond their limits posing new challenges to emergency healthcare services and critical care. The outbreak continues to jeopardize human health, social life and economy. All known human CoVs have zoonotic origins. Recent detection of SARS-CoV-2 in pet, zoo and certain farm animals has highlighted its potential for reverse zoonosis. This scenario is particularly alarming, since these animals could be potential reservoirs for secondary zoonotic infections. In this article, we highlight interspecies SARS-CoV-2 infections and focus on the reverse zoonotic potential of this virus. We also emphasize the importance of potential secondary zoonotic events and the One-Health and One-World approach to tackle such future pandemics.

## Introduction

The novel coronavirus (2019-nCoV) was first detected in humans suffering from an atypical fatal pneumonia in early December 2019 in Wuhan, China. Although initial zoonotic transmission was suggested via the Huanan seafood market that also traded live wild animals [1, 2], the role of this market in spreading this virus remained unclear. Isolation of the virus from environmental samples collected from this market suggested the possibility of its crossing the species barrier from animal(s) to humans. On 7 January 2020, the 2019-nCoV was isolated from the nasal swab of a human patient [3], and re-named as SARS-CoV-2 by the *Coronaviridae* Study Group (CSG) of the International Committee on Taxonomy of Viruses (ICTV; [4]). The World Health Organization (WHO) named the resulting disease as COVID-19, an acronym derived from the Coronavirus Disease of 2019. On 30 January 2020, the WHO declared the coronavirus outbreak as a Public Health Emergency of International Concern. With its rapid spread across continents, the WHO categorized the COVID-19 as a “Pandemic” on 11 March 2020. As of 24 August 2020, COVID-19 had caused over 816,535 deaths with more than 23,800,750 reported infections worldwide with a global case fatality rate (CFR) of 3.43% (covidvisualizer.com). Many countries including the USA and Canada have declared the COVID-19 epidemic as a “National Emergency” and have mobilized extra financial and public health resources to combat it.

## Animal coronaviruses

**Figure 1.**
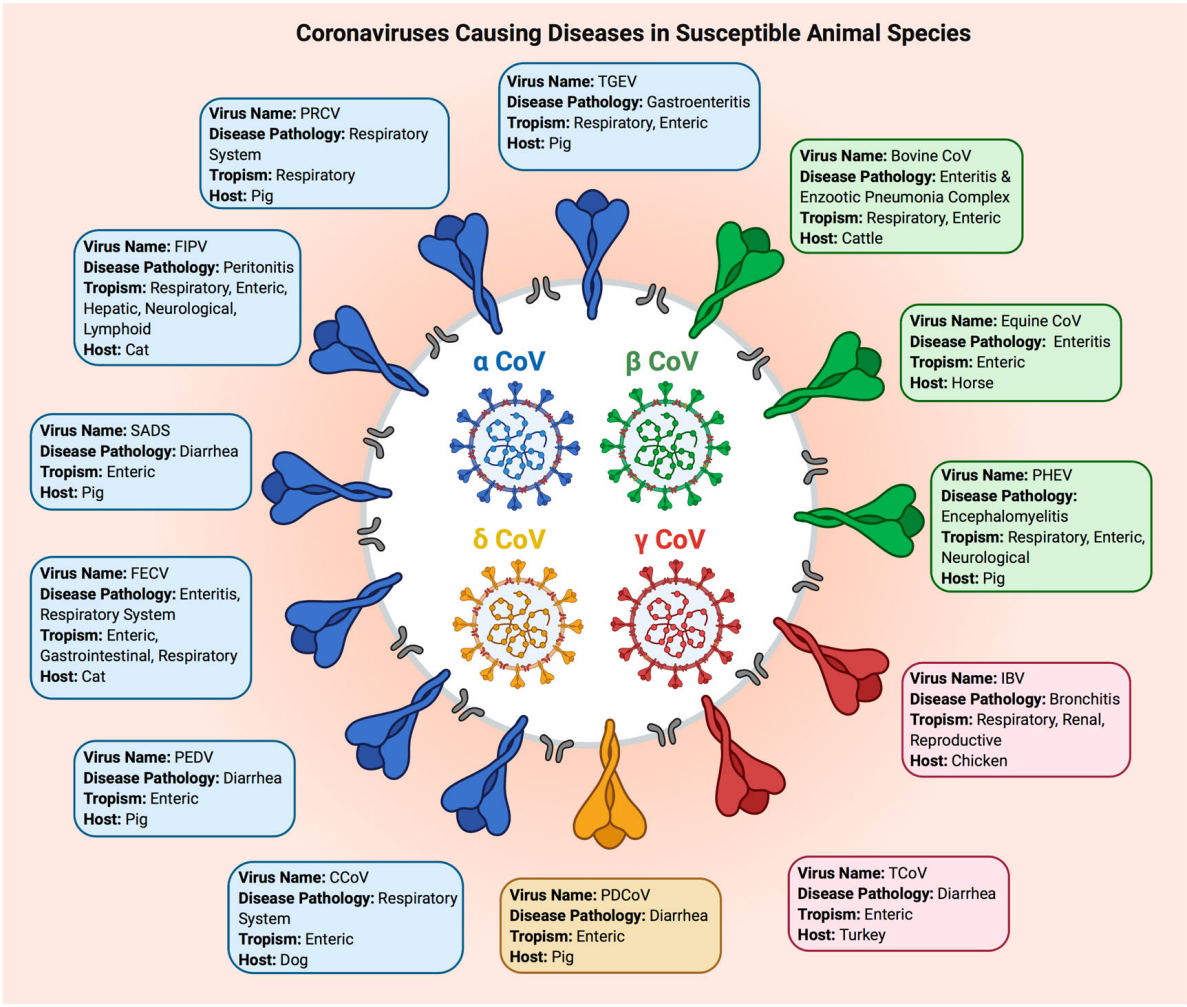
Selected important coronaviruses causing diseases in animal species. The figure shows different coronaviruses, their genera, main clinical symptoms, host species, and tissue/organ tropism. PEDV: Porcine Epidemic Diarrhea Virus; TGEV: Transmissible Gastroenteritis Virus; PRCV: Porcine Respiratory Coronavirus; FIPV: Feline Infectious Peritonitis Virus; FECoV: Feline Enteric Coronavirus; CCoV: Canine Coronavirus; PDCoV: Porcine Delta Coronavirus; TCoV: Turkey Coronavirus; IBV: Infectious Bronchitis Virus; PHEV: Porcine Hemagglutinating & Encephalomyelitis Virus; Equine CoV: Equine Coronavirus; BCoV: Bovine Coronavirus; Severe Acute Diarrhea Syndrome Coronavirus: SADS-CoV.

Coronaviruses (CoVs) are subdivided into four genera: α, β, γ and δ. Since late 1930s, different animal CoVs have been isolated from various infected animals and avian species including rodents, cattle, pigs, cats, camels, bats, dogs and and chickens and turkeys [5–7]. The viruses cause respiratory, reproductive, gastrointestinal, hepatic, neurological and/or other systemic pathologies in a wide range of animal and avian species (Figure 1; [5–7]). Phylogenetic analyses of two porcine CoV, Porcine Respiratory Virus (PRCV) and Transmissible Gastroenteritis Virus (TGEV) revealed that the former originated from the latter through a deletion in a part of the Spike (S) protein that altered the tissue tropism of the virus from the gastrointestinal tract to the respiratory tract [5–10]. The deletion also resulted in reduced virulence of the new variant. It is noteworthy that all animal (and human) coronaviruses belong to the α and β genera with the exception of one, the Porcine Delta CoV (PDCoV; Figure 1). The PRCV, porcine enteric diarrhea virus (PEDV) and the more recently emerged swine acute diarrhea syndrome coronavirus (SADS-CoV) [7] cause significant economic losses to swine industry. The CoVs belonging to the γ genus mainly infect domestic birds. The avian infectious bronchitis virus (IBV), the first CoV detected in 1937 in chickens [7–9], affects the respiratory, renal and reproductive systems of chickens. Delta CoVs mainly infect wild birds, including bulbul coronavirus and sparrow coronavirus [6, 7, 10].

## Zoonotic origins of human coronaviruses

**Figure 2.**
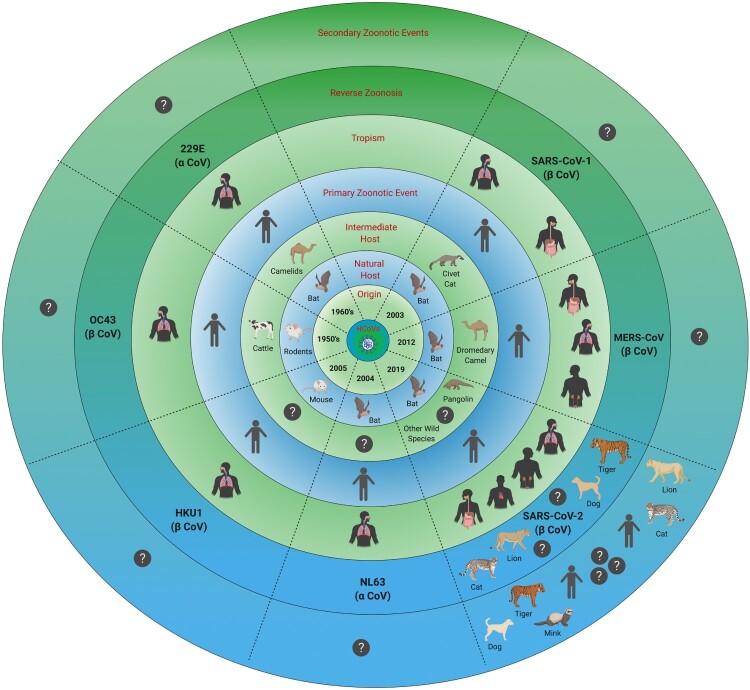
Human coronaviruses. The figure shows seven human coronaviruses, their origins, natural reservoirs, intermediate hosts, tissue/organ tropism and reverse zoonosis along with primary and potential secondary and tertiary zoonotic events. An interrogative sign (?) indicates unknown or unidentified.

At present, seven species of CoVs are known to infect humans (Figure 2). Four of these human CoVs (HCoVs) namely 229E, OC43, NL63 and HKU1 are endemic, and cause 15-30% cases of common cold in humans, mostly during winter and early spring [5, 8–13]. The 229E strain B814 was the first HCoV identified in 1966 in the nasal swab of a human patient, who had contracted “common cold” [5, 8–15]. HCoV-OC43 was isolated in 1967 from the organ culture from an infected human [5, 8, 9, 13–15]. Comparative phylogenetic analyses of human and animal CoVs suggest that 229E and OC43 jumped their animal reservoirs (bats and cattle, respectively) to infect humans within the last 200 years ([8, 9, 11–15]; Figure 2). HKU1, isolated in 2004 from an elderly patient in Hong Kong, is believed to have originated from mice [5, 8, 9, 14, 15], while NL63, isolated in the same year from a seven-month old child in the Netherlands, jumped from bats to an unidentified intermediate host before crossing species barrier to humans ([5, 13, 14]; Figure 2). Within the last two decades, three β-CoVs have jumped from animal reservoirs directly or through intermediate hosts to cause severe diseases in humans; these are Severe Acute Respiratory Syndrome coronavirus (SARS-CoV-1) [14–21], Middle East Respiratory Syndrome coronavirus (MERS-CoV) [19–20] and the most recent being SARS-CoV-2 causing COVID-19 in humans ([4, 12, 13, 20, 21]; Figure 2). The first case of SARS, presenting with atypical pneumonia, was documented in late 2002 in the Guangdong province of China [5, 14, 17, 18, 20]. The SARS epidemic caused 8,096 reported cases with 774 deaths, in many countries of the world [17–20]. MERS, first reported in Saudi Arabia in the year 2012, caused only 2521 cases with 866 deaths. It proved more fatal than SARS-CoV-1 with CFR 34% versus 11% for MERS [17–20, 22]. SARS-CoV-1 evolved to jump the species barrier through intermediate hosts “masked palm civets and raccoon dogs” causing epidemic, while MERS-CoV jumped through the intermediate host “dromedary camels” to infect humans ([5, 14, 19, 20]; Figure 2). MERS cases still occur periodically, likely resulting from occasional spill-over from the intermediate host “dromedary camels” [5, 12–14, 18–20, 22].

SARS-CoV-2 is more contagious compared to SARS-CoV-1 and MERS-CoV [5, 12, 20]. The basic reproductive number (R0 value) of SARS-CoV-2 is still controversial. The early estimates of R0 value for SARs-CoV-2 ranged from 2.2–3.1 [23, 24]. The recent estimates, however, indicate that the number of SARS-CoV-2 infected individuals doubles every 2.4 days during the early epidemic, and R0 is likely to range from 4.7–6.6 [25–28]. In a recent report, R0 of 3.54 was estimated for SARS-CoV-2 in the early outbreak in Wuhan, China [29] that is much higher than those reported for SARS-CoV-1 and MERS; the authors identified two key features of the outbreak in Wuhan: high covertness and high transmissibility of the SARS-CoV-2 infections [29]. Effective multipronged interventions and mitigation measures have considerable positive effects on controlling the outbreak i.e. decreasing the R0 value [29]. The higher transmission rates of the virus imply that short-term control measures (lockdowns, self-isolation and quarantines) have to be replaced by long-term measures through effective prophylactic vaccine [25, 27–29].

Although bats are considered a natural reservoir of more than 5,000 diverse CoVs, only about 500 of them have been identified [5, 12, 14, 21, 30]. Based on comparative phylogenetic analyses, bats are considered the most likely natural reservoirs of SARS-CoV-1 and MERS-CoV. Bats have co-evolved with viruses for millions of years. The co-evolution has led bats to constitutively express higher levels of type I and type III interferons in their body cells. The interferons are known for inducing a large variety of antiviral genes, called Interferon-stimulated genes (ISG), which limit viral replication [5, 13, 16]. On the other hand, bats induce a very subdued tissue destructive and pathogenic inflammatory response to viruses. Viral infections in bats do not produce excessive amounts of cytokines and other pro-inflammatory mediators that are responsible for causing disease [5, 13, 16]. Furthermore, bats also exert attenuated responses to several pathogen-associated (e.g. viral RNA) and danger-associated molecular patterns (e.g. cytosolic DNA). It is noteworthy that a subset of ISG, such as RNA-dependent adenosine deaminases (ADARs) and apolipoprotein B mRNA editing enzyme catalytic polypeptide-like (APOBEC) family genes, promote mutations in the viral RNA leading to the emergence of novel viral strains, and at the same time attenuating host’s innate immune responses to viral RNA [31, 32]. Thus, the persistence of viral infections, increased RNA editing, and longer life span of bats increase the probability of viral mutations and recombination events, which potentially contribute to the emergence of infectious and pathogenic CoV strains for other species. It is not surprising that bats act as reservoirs for CoVs as well as for many other highly pathogenic viruses, including rabies and hemorrhagic fever viruses [5, 11–14].

Unlike in bats, CoV infections in humans show a wide spectrum of disease severities. They may be asymptomatic, mild or severe, and may result in death [11, 16]. The severity of the disease depends upon the degree to which the infected host can mount an antiviral response without eliciting a self-destructive inflammatory response [11, 12]. The factors that determine nature of the host response could be both of viral and host origins [11, 16, 33]. A viral strain that has been circulating in the host and hence adapted to it, will be less pathogenic. Host’s genetic propensity to exert excessive inflammatory response and the presence of pre-existing chronic inflammatory conditions (e.g. obesity, old age, cardiovascular disease, etc.) are known to aggravate the disease severity in COVID-19 patients [11, 12, 16]. Recent studies have shown that a prior exposure of humans to other Coronaviruses (SARS and/or “common cold”-causing ones), induces cross-reactive antibodies and memory T cells that protect them when they contract SARS-CoV-2 infection [34]. About 20–50% of humans were shown to have such protection; they are likely to have no or mild clinical symptoms upon infection with SARS-CoV-2.

Similar to other RNA viruses, CoVs are prone to random mutations as they possess a relatively large RNA genome with a limited proof-reading activity. The mutation rate in SARS-CoV-2 (<25 mutations/year) is, however, less than that of influenza A virus (≥50 mutations/year; [35, 36]). Recent studies suggest that SARS-CoV-2 continues to evolve as new mutation hotspots/ mutated strains of this virus are emerging in different parts of the world [35, 37]. The ultimate aim of viruses such as SARS-CoV-2 is to attain a point of equilibrium with their host and become endemic without causing high mortality in a susceptible host population. European, North American and Asian viral strains may possibly coexist, each characterized by a different mutation patterns in various viral genes, including RNA-dependent – RNA polymerase (RdRp) and Spike [35, 37]. The characterization of viral mutations is important not only for understanding viral drug resistance, immune evasion and pathogenesis-related mechanisms, but also for the development of vaccines, and designing antiviral drugs and diagnostic assays [35, 37].

## SARS-CoV-2 interspecies infections

Since the initial reports of mysterious pneumonia (COVID-19 pneumonitis) from Wuhan in China, there has been significant debate on why SARS-CoV-2 infects humans, and several hypotheses have been proposed. Through their Spike (S) protein, all CoVs bind their receptors on host cells and initiate the infection. The cleavage of S protein two segments (S1 and S2) by a host protease is essential for the infection. S1 carries the receptor-binding domain (RBD), which determines its specificity for the viral receptor on host cells. Mutations in S protein of SARS-CoV-2, due to natural selection, might have resulted in its higher affinity for human angiotensin converting enzyme-2 (ACE2) receptors [21]. The receptors are expressed in nasal mucosa, bronchus, lung, heart, esophagus, kidney, stomach, urinary bladder, testes and ileum [38], making these human organs highly vulnerable to infection with SARS-CoV-2.

It has been well known that the amino acid sequences of the cleavage peptide at the S1 and S2 junction determine the peptide’s susceptibility to different host proteases, and contribute to viral tissue tropism, host range and pathogenesis. As mentioned above, the surface unit S1 binds to a cellular receptor, while the transmembrane unit S2 facilitates fusion of the viral membrane with cellular membrane. SARS-CoV-2 has acquired a polybasic cleavage site containing arginine residues at the S1/S2 junction. The acquisition is an important evolutionary step necessary for the viral cross over to humans [21, 39, 40]. The polybasic site is important for several reasons. First, the site enables cleavage of S protein into S1 and S2 segments by furin, a ubiquitous protease, facilitating rapid viral dissemination from lungs to other tissues. Second, residues in the cleavage site influence 3D structure of S-protein, its furin binding affinity and alter viral virulence. Third, proteolytic cleavage of SARS-CoV-2 S-protein by furin is a prerequisite for subsequent activation (cleavage) of S2 by another host cell protease, the Transmembrane Serine Protease 2 (TMPRSS2), an essential step for viral entry into a host cell. Last but not the least, the SARS-CoV-2 S1/S2 cleavage site is identical to the furin-cleavage site present in the human epithelial sodium channel α-subunit (ENaC-α) [41]. In distal lung airways, ENaC is known to play a key role in controlling fluid reabsorption at the air–liquid interface. The proteolytic activation of ENaC-ɑ by furin is required for the functional activation of this channel. It has been postulated that the viral S protein competes with ENaC-α for furin engagement leading to poor fluid reabsorption in the lungs of COVID-19 patients [41].

The importance of a polybasic furin cleavage site for tissue tropism and viral pathogenicity has been well documented in avian influenza A viruses. Analogous to the SARS CoV-2 S protein, the hemagglutinin (HA) of the Influenza viruses needs cleavage into HA1 and HA2 segments for their entry into host cells [42]. The viruses have been classified into low – or high-pathogenic avian influenza viruses (LPAIVs or HPAIVs, respectively) [42]. The LPAIVs cause asymptomatic or mild infections whereas HPAIVs cause severe systemic infections in poultry with high mortality. Remarkably, the LPAIVs possess a monobasic (usually a single arginine) cleavage site at the HA1/ HA2 junction. The site is cleaved by trypsin and trypsin-like proteases, whose expression is restricted to avian respiratory and intestinal tract tissues [42]. On the other hand, the HA of HPAIVs contains a polybasic motif, which is cleaved by ubiquitously expressed proprotein convertases such as furin leading to severe systemic infections with high mortality. Interestingly, HPAIV strains emerge from LPAIV by insertion of multiple basic amino acids at the cleavage site loop. Thus, the acquisition of a polybasic cleavage site is an essential feature for the evolution of HPAIV from LPAIV.

Given the fact that SARS-CoV-2 is the only virus among SARS-related viruses whose S protein contains a peculiar furin cleavable polybasic cleavage site, the feature most likely contributes to the viral transmissibility, zoonotic potential, wide-spread tissue distribution and a wide spectrum of clinical symptoms. A recent study suggests that a D614G mutation in the S-protein, which renders S protein more susceptible to cleavage by furin and TMPRSS2, is linked to a higher transmission rate of SARS CoV-2 [43, 44]. In addition to SARS CoV-2, many other human viruses of medical importance such as HIV-1, measles virus, respiratory syncytial virus, and Ebola virus require furin for cleavage and activation of their receptor-binding surface glycoproteins [42]. Unfortunately, because of its essential role in a wide array of cellular functions, furin cannot be targeted for devising anti-viral strategies.

SARS-CoV-2 shares 96.2% identity at the nucleotide level with RaTG13, a CoV detected in horseshoe bat species, *Rhinolophus sinicus;* this virus, however, has not been detected in humans [21]. Although horseshoe bat has been proposed to be the natural host of SARS-CoV-2, no direct evidence on the existence of SARS-CoV-2 infection in the bats is documented to date. Despite the homology, the RaTG13 RBD is very divergent from that of the SARS CoV-2. Possibly due to selective pressure, the SARS-CoV-2 RBD, after evolving in horseshoe bats, has evolved further in a non-bat intermediate animal species before its zoonotic transfer to humans. In this regard, it is interesting to note that the SARS CoV-2 RBD is very similar to the one found in the SARS-related β-CoVs of Malayan pangolin [45–48], an endangered mammalian species. The viral sequences were found in archived samples of pangolins, who, similar to COVID-19 patients, had exhibited clinical symptoms such as cough and shortness of breath [46–48]. Moreover, there was evidence of vertical transmission of the SARS-related β-CoVs in pangolins, suggesting virus circulation in this natural population [48]. However, testing of throat and rectal swabs from 334 pangolins sampled from the market’s upstream supply chain, did not yield any positive PCR results for this CoV’s nucleic acid sequences [46], suggesting that positive samples from pangolins in the market may have resulted from exposure to infected humans, wildlife or other animals. Thus, the existing evidence does not conclusively support the hypothesis that RaTG13 or pangolin β-CoV is the immediate parental virus of SARS-CoV-2. A recent phylogenetic analysis indicates a strong purifying selection around SARS-CoV-2 RBD and other genes among bat, pangolin, and human CoVs through recombination event(s) with CoVs from pangolins giving SARS-CoV-2 surface protein the ability to infect human cells [49]. It is likely that SARS-CoV-2’s RBD became optimized through pre-adaptation in pangolins for binding to human ACE2 receptors with high affinity. Biophysical and structural evidence suggests that the SARS-CoV-2‘s RBD is likely to bind human ACE2 with 10–20-fold higher affinity than that of SARS-CoV-1 [21, 45, 50–52]. To date, there is not enough scientific evidence to accurately explain the original route of SARS-CoV-2 transmission to humans, which may involve an un-identified intermediate host. Further investigations are required to establish an evolutionary pathway of SARS-CoV-2 in bats, pangolins and/or other mammals. Since the cat ACE2 gene is the closest to the human’s among non-primate animals, and can potentially bind with both SARS CoV-1 and SARS CoV-2 S proteins [53], it is important to monitor the presence of SARS-CoV-2 infection in cats because human patients with COVID-19 may potentially transmit this virus to cats.

The current concept is that parental viruses of HCoVs are archetypically non-pathogenic in their natural reservoir host(s) but become pathogenic after their transmission to a new host species. With further evolution, the viruses adapt to the new host and become more transmissible and less pathogenic [5, 43, 44]. It is therefore possible that a progenitor of SARS-CoV-2 jumped into humans and adapted from being more virulent to a more transmissible viral strain responsible for the current pandemic [21, 43, 44]. It is important to note that SARS-CoV-2 shows pathogenicity less than that of the SARS-CoV-1 and MERS-CoV but a transmissibility potential similar to or greater than that of the – community-acquired HCoVs (NL63, 229E, OC43, and HKU1) [5]. It is highly probable that the virus in humans will continue to evolve on this line and adapt further to humans and become less pathogenic unless intervened by therapeutics and/or prophylactic vaccines.

The dynamics of viral quasi-species resulting from genetic variation, competition and selection might have played a role in SARS-CoV2 adaptation to infect human cells. The quasi-species phenomenon has been reported for several RNA viruses including SARS CoV-1 and MERS-CoV [54, 55]. A recent genomic analysis of 103 viral isolates suggests that SARS-CoV-2 has evolved into S and L types [56], although the clinical significance of these two types is uncertain. It is possible that one strain type is more pathogenic than the other. Several genotypes, antigenic types and pathotypes of SARS-CoV-2 are possibly circulating among human populations. Future studies involving a large number of viral isolates might help to characterize genotypes and potential antigenic variants of this virus. The characterization will have important implications for the development of therapeutics, vaccines and diagnostic approaches.

## SARS-CoV-2: experimental infections

These infections are important to investigate pathogenetic mechanisms of the virus, host immune response and efficacy of different drugs and vaccines. In this regard, structural modelling of the SARS-CoV-2 S protein and ACE2 receptors indicates that the virus could bind and perhaps infect bat, civet, monkey and swine cells [57]. However, host range projections based on modelling are sometimes not correct and require *in-vivo* studies in animals to determine the host range of the virus [30]. Based on structural modelling of these proteins, Asian and African primates are likely more susceptible to SARS-CoV-2 compared to the South and Central American primates [51]. On the bases of viral genome sequence analyses, and structural and biochemical studies, it seems more likely that the SARS-CoV-2’s RBD binds with high affinity to ACE2 from humans and ferrets [52, 57–59, 60–62]. In concordance with these findings, Macaques (Macaca fascicularis and Macaca mulatta), ferrets and Syrian golden hamsters were not only found to be highly susceptible to experimental infection with SARS-CoV-2 but these animals could also transmit the infection to their cage mates (Table 1; [51, 57–59]). Macaques develop respiratory signs very similar to those of COVID-19 patients [51] whereas in Syrian golden hamsters and ferrets, SARS-CoV-2 infections cause none or mild respiratory signs [57–59]. These animal species appear to be suitable animal models for investigations on COVID-19 [51, 58, 59]. Recently, North American deer mice (Peromyscus maniculatus) was also shown to be susceptible to experimental infection with SARS-CoV-2 [60]. ACE-2 receptors of these mice possess key amino acid residues that allow cell binding to SARS-CoV-2 S protein. However, the risk of reverse zoonosis and/or potential role of this animal species as a reservoir for SARS-CoV-2 needs further investigation. Initial findings suggest that chickens, ducks, turkeys and pigs are refractory to infection by SARS-CoV-2 under experimental conditions [57].

**Table 1.** Animal Species Susceptibility to SARS-CoV-2.

Species	Susceptibility	Infection type	Clinical signs	Transmission	References
Lion/Tigers	High	Natural	None or mild (mild respiratory disease and dry cough)	Animal to animal, human to animals; virus shed in feces and perhaps respiratory secretions	[65, 66, 70]
Dogs	Low	Natural/Experimental	None or very mild (respiratory signs possible; comorbidities may increase the susceptibility or severity of signs)	None reported; dogs may shed virus in nasal secretions	[57, 64–70]
Domestic Cats	High	Natural/Experimental	None or mild (mild respiratory signs such as sneezing, transparent ocular discharge, and lethargy; presence of other respiratory pathogens or comorbidities may increase the severity of the signs)	Cat to cat; cats shed virus in their nasal secretions and feces; air-borne transmission reported among cage mates	[53, 57, 64–66, 68, 70–73]
Poultry (chickens and ducks)	None	Experimental	None	None	[57, 80]
Pigs	None	Experimental	None	None	[57, 80]
Ferrets	High	Experimental	None or mild (sneezing, elevated temperature, reduced activity and occasional cough)	Ferret to ferret; ferrets shed virus in nasal secretions, saliva, urine and feces; air-borne transmission among cage mates reported	[52, 57, 59, 80]
Rhesus Macaques (*Macaca fascicularis* and *Macaca mulatta*)	High	Experimental	Moderate signs (irregular respiratory pattern, reduced appetite, hunched posture, pale appearance, dehydration, elevated temperature and weight loss as well as pulmonary infiltrates evident on lung radiograph)	Animal to animal; virus is shed in saliva, nasal secretions and feces	[51]
Fruit Bats (*Rousettus aegyptiacus*)	High	Experimental	None or mild (rhinitis)	Bat to bat; fruit bats shed virus via respiratory, oral and fecal routes	[80]
Farmed Minks	High	Natural	None or moderate to severe signs (gastrointestinal and respiratory signs, pneumonia and increased mortality rate)	Human to mink, mink to mink, mink to cat possible, mink to human possible; minks shed virus in respiratory and oral secretions as well as in feces	[66, 74]
Golden Syrian Hamsters	High	Experimental	Mild (progressive weight loss, lethargy, ruffled furs, rapid breathing and hunched back posture)	Hamster to hamster; hamsters shed virus in respiratory secretions and feces	[58]
Deer Mice (*Peromyscus maniculatus*)	High	Experimental	None or very mild (ruffled fur)	Mice to mice; mice shed virus in nasal secretions, saliva and feces	[60]

Recently, it was shown that the bat intestinal organoids (more specifically enteroids) were susceptible to infection with SARS CoV-2 and showed cytopathic effects [63]. This is not surprising considering 80.5% amino acid sequence similarity between human and horseshoe bat ACE-2 receptors. The establishment and characterization of the bat enteroids that simulate the cellular composition of the bat intestinal epithelium might allow rapid and robust isolation and study of SARS-CoV-2 isolates or its progenitor(s) with higher efficiency than Vero E6 cells; later being the most commonly used cell line for SARS-CoV-2 isolation and characterization [63]. In addition, the authors also reported active SARS-CoV-2 replication in human enteroids, suggesting that human intestinal tract might be an additional route for viral transmission [63]. Whether the intestinal epithelial cells are primarily infected with SARS-CoV-2 via the oral–fecal route or enteric infection occurs post-respiratory infection needs to be investigated.

## Reverse zoonosis (Zooanthroponosis) and secondary zoonosis

Sporadic detection of natural SARS-CoV-2 infections together with successful experimental infections of certain animals raises concerns about reverse zoonosis (also termed as zooanthroposis: transmission of the infection from humans to animals) as well as about secondary zoonotic events (transmission of the infection from animals back to humans). Several cases of dogs, cats and zoo animals have tested positive for SARS-CoV-2, mostly as a result of close contact with infected humans (Table 2; [64–66]). However, the occurrence of natural infections in these animal species has not been ruled out. There is a limited information available on clinical manifestations of SARS-CoV-2 in animals. The existing evidence suggests that the clinical manifestations may range from covert infections to symptomatic disease with signs that may include coughing, sneezing, respiratory distress, nasal discharge, ocular discharge, vomiting or diarrhea, fever, and lethargy, etc. (Table 1). The detection of asymptomatically prevalent infections in companion animals, and estimation of their proportionate contribution in the spread of SARS-CoV-2 to humans and other animal species, if any, requires further investigations.

**Table 2.** Reverse zoonosis cases of SARS-CoV-2 reported in animals.

Case #	MM/DD/YYYY	Possible source of infection^d^	Animal	Region/Country	References
1	02/27/2020	Pet Owner	Dog ^ab^	Hong Kong	[66–68, 70]
2	03/18/2020	Pet Owner	Dog^abc^	Hong Kong	[66–68, 70]
3	03/18/2020	Pet Owner	Cat^a^	Belgium	[66, 68, 70]
4	03/27/2020	Zoo Employee	Tiger^a^	NY, USA	[64–66, 70]
5	03/27/2020	Zoo Employee	Lion^a^	NY, USA	[64–66, 70]
6	03/30/2020	Pet Owner	Cat^ab^	Hong Kong	[66, 70]
7	04/01/2020	Pet Owner	Cat^ab^	NY, USA	[65, 66]
8	04/02/2020	Pet Owner	Cat^b^	China	[66]
9	04/04/2020	Zoo Employee	Tiger^a^	NY, USA	[64–66, 70]
10	04/06/2020	Pet Owner	Cat^ab^	NY, USA	[65, 66]
11	04/15/2020	Zoo Employee	Lion^a^	NY, USA	[66, 70]
12	04/22/2020	Pet Owner	Cat^a^	NY, USA	[65, 66]
13	04/22/2020	Pet Owner	Cat^a^	NY, USA	[65, 66]
14	04/26/2020	Farm Worker	Mink^abc^	The Netherlands	[66, 74]
15	04/28/2020	Pet Owner	Dog^a^	NC, USA	[65, 66]
16	05/01/2020	Pet Owner	Cat^a^	France	[66]
17	05/08/2020	Pet Owner	Cat^a^	Spain	[66]
18	05/08/2020	Farm Worker	Mink^abc^	The Netherlands	[66, 74]
19	05/12/2020	Pet Owner	Cat^a^	France	[66]
20	05/13/2020	Pet Owner	Cat^a^	Germany	[66]
21	05/15/2020	Farm Workers/Infected Minks	Cat^ab^	The Netherlands	[66]
22	05/15/2020	Human	Dog^b^	The Netherlands	[66]
23	05/18/2020	Pet Owner	Cat^a^	Russia	[66]
24	05/21/2020	Human	Cat^a^	Spain	[66, 73]
25	05/25/2020	Human	Cat^b^	The Netherlands	[66]
26	05/27/2020	Pet Owner	Dog	NC, USA	[65, 66]
27	06/01/2020	Pet Owner	Dog^ab^	NY, USA	[65, 66, 69]
28	06/01/2020	Pet Owner	Cat^a^	MN, USA	[65, 66]
29	06/02/2020	Farm Worker	Mink^abc^	The Netherlands	[66]
30	06/02/2020	Pet Owner	Dog^a^	NY, USA	[65, 66]
31	06/03/2020	Pet Owner	Cat^ac^	MN, USA	[65, 66]
32	06/04/2020	Pet Owner	Cat^a^	IL, USA	[65, 66]
33	06/24/2020	Pet Owner	Dog^b^	NY, USA	[65, 66]
34	06/24/2020	Pet Owner	Dog^b^	NY, USA	[65, 66]
35	07/01/2020	Pet Owner	Dog^a^	GA, USA	[65, 66]
36	07/08/2020	Pet Owner	Dog^a^	TX, USA	[65, 66]
37	07/08/2020	Pet Owner	Cat^a^	GA, USA	[65, 66]
38	07/09/2020	Pet Owner	Dog^a^	SC, USA	[65, 66]
39	07/15/2020	Pet Owner	Dog^a^	AZ, USA	[65, 66]
40	07/21/2020	Pet Owner	Cat^a^	TX, USA	[65, 66]
41	07/22/2020	Pet Owner	Cat^b^	UT, USA	[65, 66]
42	07/22/2020	Pet Owner	Cat^b^	UT, USA	[65, 66]
43	07/22/2020	Pet Owner	Dog^b^	UT, USA	[65, 66]
44	07/22/2020	Pet Owner	Dog^b^	WC, USA	[65, 66]
45	07/22/2020	Pet Owner	Dog^b^	WC, USA	[65, 66]
46	07/22/2020	Pet Owner	Dog^b^	NC, USA	[65, 66]
47	08/03/2020	Pet Owner	Dog^a^	LA, USA	[65, 66]
48	08/11/2020	Pet Owner	Dog^a^	NC, USA	[65, 66]
49	08/12/2020	Pet Owner	Cat^b^	NY, USA	[65]
50	08/17/2020	Farm Worker	Mink^a^	Utah, USA	[65]
51	08/17/2020	Farm Worker	Mink^a^	Utah, USA	[65]

^a^ Positive by RT-PCR.

^b^ Positive by virus neutralizing antibodies.

^c^ Positive on virus isolation in cell culture.

^d^ SARS-CoV-2 positive (symptomatic or asymptomatic) pet owners, animal caretakers or farm workers most likely transmitted the virus to animals.

A report from Hong Kong in February 2020 suggested the possible presence of SARS-CoV-2 infection without any signs of illness in two dogs (Table 2; [67]); the owner of these dogs was previously diagnosed with COVID-19. The first case was a 17-year old Pomeranian breed dog, who tested positive for SARS-CoV-2 RNA by RT–PCR on multiple nasal and oral swabs. However, the virus could not be isolated from the dog’s samples; mere presence of viral RNA does not decisively confirm active infection; serological testing, virus isolation and/or serial quantification of viral nucleic acids are required to confirm the infection. Although the dog tested negative on subsequent RT–PCR testing, seroconversion was noted in the initial sample, but additional testing was not possible due to unavailability of convalescent serum; the geriatric dog passed away three days later presumably due to some other underlying health problems. It was therefore concluded that the dog either had a low-level of infection or was contaminated with SARS-CoV-2 by close contact with and/or exposure to an infected person. The second case was a two-year old German Shepherd dog, who tested positive by RT–PCR in quarantine after its owner was confirmed to have COVID-19 [67, 68]. The first dog in the US reported to test positive for SARS-CoV-2 was a six-year old male German Shepherd, whose owner had confirmed COVID-19 (Table 2; [69]). The dog tested positive in mid-April 2020 and was euthanized on 11 July 2020. Although the dog tested negative on RT–PCR for SARS-CoV-2 five days later, he did develop anti-SARS-CoV-2 antibodies indicating he had active infection. This dog showed signs of muco-purulent nasal discharge with laboured breathing and lethargy [69]. The additional signs noted were blood in the urine, clotted blood in the vomit, troubled walking and weight loss. This dog was also diagnosed with lymphoma and heart murmurs [69]. This case raised two important questions. First, what signs (if any) in this dog were due to SARS-CoV-2 infection, and second, whether comorbidities such as heart problems, or cancers, play any role in increasing the susceptibility of companion animals to SARS-CoV-2 infection. Research has shown that dogs exposed to SARS-CoV-2 could produce anti-SARS-CoV-2 antibodies without exhibiting symptoms of COVID-19 [57, 68, 70].

In March 2020, the SARS-CoV-2 RNA was detected in the feces and vomit contents of a cat by RT–PCR in Belgium (Table 2; [70]). The cat belonged to an owner who tested positive for SARS-CoV-2-. The cat exhibited transient respiratory and gastrointestinal disease one week after the owner became symptomatic for COVID-19 [70]. Since no information on virus isolation, seroconversion and blood results was available at the time of this report, it was difficult to establish an association between the cat’s clinical signs and active SARS-CoV-2 infection. On 1 April 2020, a pet cat tested positive for SARS-CoV-2 without showing any clinical signs of illness in Hong Kong after her owner was confirmed to have COVID-19 [70]. On April 05, 2020, a four-year old Malayan tigress at the Bronx Zoo in New York developed dry cough and tested positive for SARS-CoV-2 (Tables 1 and 2; [70]). Five other tigers and lions showed mild signs of respiratory illness and later recovered completely ([65, 66, 70]; Figure 2; Table 2). A zoo employee, an asymptomatic carrier of SARS-CoV-2, was deemed responsible for transmitting the virus to these zoo animals. On 22 April 2020, two domestic cats tested positive for SARS-CoV-2 in the USA, one cat contracted the infection from the virus-positive owner; the other cat presumably got infected from a covertly infected human [71]. As of 14 August 2020, 14 cases of dogs and 13 cases of domestic cats in the USA have tested positive for SARS-CoV-2 infection using RT–PCR or virus neutralization antibody tests. All these companion animals had exposure to either a covertly infected or confirmed human(s) with COVID-19 [65]. It has been shown that under experimental conditions, domestic cats are susceptible to infection with SARS-CoV-2 and can transmit the virus to other cats via droplet or short-distance aerosol ([57, 70]; Table 1); the susceptibility of cats to SARS-CoV-2 is explained by the fact that feline ACE-2 receptor differs only by three amino acids from that of humans [53]. According to a report, SARS-CoV-2 had infected cat population in Wuhan during the COVID-19 outbreak; 14.4% cat sera collected before and after the COVID-19 outbreak were positive for antibodies specific to the RBD of SARS-CoV-2 by indirect enzyme linked immunosorbent assay [72]. Among the 15 positive cat sera, 11 serum samples had various titers of SARS-CoV-2 neutralizing antibodies; the authors did not detect any serological cross-reactivity between the SARS-CoV-2 and FIPV. Recently, a large-scale study involving 500 companion animals (dogs and cats) living in COVID-19-positive and – negative households in Northern Italy reported measurable SARS-CoV-2 neutralizing antibody titers in 3.4% dogs and 3.9% cats [73]. These reports support the notion of human-to-animal transmission (reverse zoonosis or zooanthroponosis) of SARS-CoV-2.

In a recent report, minks showing signs of pneumonia and mortality were confirmed to be infected with SARS-CoV-2 at various farms in the Netherlands ([74]; Tables 1 and 2; Figure 2). It is believed that several farm workers, who developed symptoms consistent with COVID-19, transmitted the virus to the minks. It is also likely that infected minks might have transmitted the virus to some of the farm workers. The transmission was evident from similarity of viral sequences detected in infected individual to those found in minks. Additionally, seven of 24 cats at the mink farms also tested positive serologically for SARS-CoV-2-specific antibodies [66, 74], and at least one seropositive cat tested positive for the SARS-CoV-2 RNA. Although, neither the source of the virus (infected minks or infected farm workers) in cats nor transmission of the virus from cat(s) to other animals or humans could be confirmed, the possibility of interspecies transmission of the virus, i.e. primary and secondary zoonotic events cannot be ruled out.

Given the evidence in this review, it is apparent that certain CoVs have crossed, and will continue to cross host species. A strong possibility exists that SARS-CoV-2 has been transmitted from humans to farmed minks and companion and zoo animals. The consequences of such zooanthroponosis are unknown at the moment. The interspecies transmission among animals could make the pandemic control more difficult. It should, however, be noted that as of today, there is no conclusive evidence that cats, dogs, or zoo animals can transmit SARS-CoV-2 to humans. Based on the current information, the overall risk of SARS-CoV-2 transmission from companion and zoo animals to other animals or humans as well from humans to animals is low. However, uncertainty remains on how this virus will behave in various animal species. Thus, urgent and planned investigations and continuous monitoring as well as targeted proactive surveillance of specific animals and their caretakers, with known or suspected exposures to humans with COVID-19, are warranted at local and national levels.

Currently, there is not enough SARS-CoV-2 specific testing being done for companion and zoo animals. However, the situation may change in future. It is noteworthy that the USA IDEXX Laboratories have recently launched a test under the commercial name “SARS-CoV-2 (COVID-19) Real PCR Test” to test samples from companion animals [75]. Several other private and government veterinary laboratories are also in the process of developing and using serological assays and nuclic acid-based tests for SARS-CoV-2 detection in pet and zoo animals.

## The One-Health initiative and potential for secondary zoonotic events

**Figure 3.**
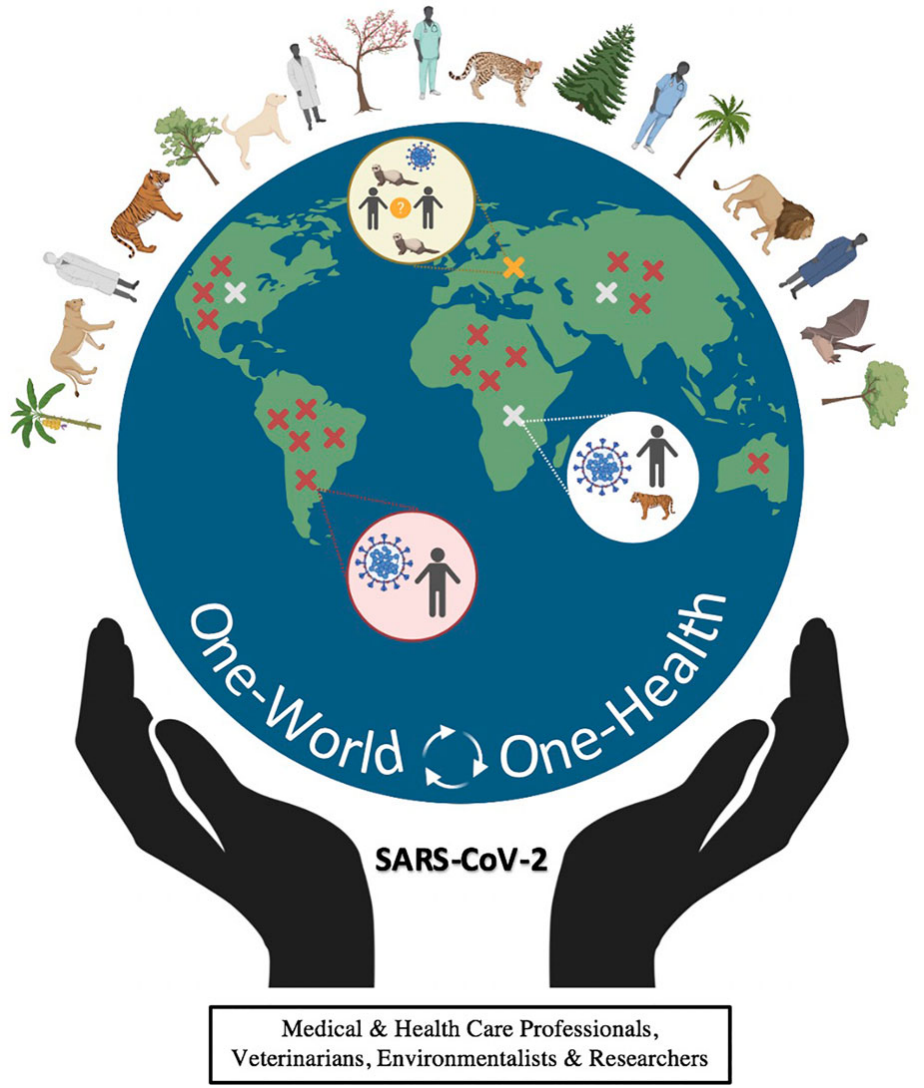
SARS-CoV-2 and the One-World – One-Health concept. It emphasizes that human health is dependent and intricately connected with that of animals (domestic and wild), birds and plants. A disturbance in the ecosystem results in human diseases (zoonotic or reverse-zoonotic). The letter X denotes a zoonotic event; the color red, white and yellow depict potential primary, secondary and tertiary zoonotic events, respectively.

The One-Health concept is based upon the premise that human health is intricately linked to, and dependent upon, the health of all the living creatures of this planet as well as of their habitats. Together they form an ecosystem, whose health ultimately affects human health (Figure 3). Therefore, it dictates that a collaborative, multisectoral, and transdisciplinary approach should be adopted at a global level for achieving the best possible health outcomes for people, animals, plants, and their shared environment. Over the last two decades, an alarming upsurge in newly emerging and certain re-emerging pathogens was observed [76]. During this period, three pathogenic CoVs have struck humans, and the probability of another one is quite certain. This scenario leaves no option other than adopting One Health-One World approach. It is worth noting that about 60% of known infectious diseases in humans are zoonotic and about 70% of emerging infectious diseases in humans are also of zoonotic origin [77]. Several factors may account for the increases in infectious diseases of zoonotic origin. They include climate change, urbanization, rapid population growth, expansion of new geographic areas, lifestyle changes, eating habits, intensive farming systems, deforestation, land use changes, increased human national and international travel, and increased movements of animals and animal products across the globe for trade. These changes affect human and animal health, and the ecosystems, resulting in rapid spread of infectious diseases. They increase the probability of SARS-CoV-2 jumping not only from animals into humans through primary and secondary zoonotic events, but also from humans to animals through reverse spread. They also enhance the potential for emergence of new genotypes, pathotypes and/or antigenic types of SARS-CoV-2. Because of the ability of the virus to infect multiple host species, it could be quickly shipped via asymptomatic human carriers, infected animals and/or food products from one location to the other.

The World Organization for Animal Health (Office International des Epizooties; OIE), the WHO and Food and Agriculture Organization (FAO) of the United Nations, the United States Department of Agriculture (USDA) and Center for Disease Control and Prevention (CDC), and many other global and local organizations are advocating and promoting the One-Health for One World approach. The occurrence of global epidemics such as HPAI, Ebola, SARS, MERS, and recently COVID-19 [33, 76–79] has provided stimulus to further strengthen the One-Health programme, especially in developing nations, and leveraging the expertise of veterinarians to support global public health community [79].

The One-Health approach recommends that veterinarians, medical doctors and human and animal health specialists be involved in inter-disciplinary collaboration to fight against not only COVID-19 but also against other newly emerging pathogens, which constantly threaten human and animal health as well as food safety and security. Adopting a One-Health strategy will not only help in developing and implementing effective strategic planning, policies, and procedures leading to timely disease agent detection and prevention but also in a coordinating emergency response and a solid contingency plan for future pathogens. The increased awareness on zoonotic and reverse zoonotic potential of SARS-CoV-2, and their mitigation measures should be communicated to the general public. The COVID-19 pandemic has also highlighted the importance of local provisions of essentials (e.g. food, medical equipment, and lifesaving drugs), and reduced dependency on global infrastructure. Veterinarians, microbiologists, epidemiologists and animal biologists might help to predict the emergence and potential source(s) of future outbreaks of infectious diseases. Surveillance of wild, food and domestic animal population is the key to preventing SARS-CoV-2 from establishing itself in another animal species, especially in companion and food animals. Future investigations on reverse zoonotic potential of SARS-CoV-2 should focus on disease transmission routes, prevalence, pathogenesis, and prevention mechanisms. It is unlikely that SARS-CoV-2 will be the last coronavirus to jump species barrier infecting human(s) and other animal species.

The lessons learned from COVID-19 pandemic could be used to create awareness for strengthening the veterinary and medical profession on modern grounds. This will aid veterinary professionals to effectively contribute to not only animal but also to public health at local, national, and global levels. The collective knowledge found, resources developed, and capacity built could be used for a more collaborated, rapid and effective emergency response that keeps in check the future spread of emerging epidemics at a both national and international level.

## Conclusions

SARS-CoV-2 is a zoonotic disease that has crossed species barrier from its reservoir bat host, and infected humans via an unknown intermediate animal species. The disease, declared as a pandemic, has caused a great loss to human life and economy, and has disrupted our routine social life. The virus has clearly shown the potential of reverse zoonosis. During the last six months there have been consistent reports of the viral infections in companion, zoo and certain farm animals. The specter of primary and secondary zoonotic events, although not yet confirmed, has also been raised. We should continue to adhere to traditional measures of controlling the pandemic (lockdowns, physical distancing, testing and isolating the infected individuals, increased personal hygiene and protection of vulnerable) until effective therapies and vaccines are developed. The recommendations of the One-Health One-World should be implemented to prevent the emergence of future epidemic(s) and pandemic(s).

## Contributors

KM, SA, IM, HK MAK, NM, SOA, MAI, RM, AA and XZ wrote the manuscript with input from KM and SA. SHA, UC, MUZ, MA, AA and XZ provided intellectual input, did literature search and proofread the article. All authors revised the manuscript and approved the final report.
